# A Mobile Game to Support Smoking Cessation: Prototype Assessment

**DOI:** 10.2196/games.9599

**Published:** 2018-06-07

**Authors:** Bethany R Raiff, Nicholas Fortugno, Daniel R Scherlis, Darion Rapoza

**Affiliations:** ^1^ Health and Behavioral Integrated Treatments Research Unit Department of Psychology Rowan University Glassboro, NJ United States; ^2^ Playmatics, LLC New York, NY United States; ^3^ Entertainment Science, Inc Durham, NC United States

**Keywords:** smoking, smoking cessation, contingency management, mobile apps, virtual rewards, health games, video games, digital games, carbon monoxide, incentives

## Abstract

**Background:**

Cigarette smoking results in an estimated seven million deaths annually. Almost half of all smokers attempt to quit each year, yet only approximately 6% are successful. Although there are multiple effective interventions that can increase these odds, substantial room remains for improvement. One effective approach to helping smokers quit is contingency management, where quitting is incentivized with the delivery of monetary rewards in exchange for objective evidence (eg, exhaled carbon monoxide levels) of abstinence.

**Objective:**

We assessed the feasibility and promise of *Inspired*, a contingency management mobile app for smoking cessation that uses game-based rewards to incentivize abstinence from smoking instead of the monetary (or material) rewards typically used. We sought participant feedback and limited objective data on: the features and design of *Inspired*, interest in using *Inspired* when it becomes available, the likelihood of *Inspired* being an effective cessation aid, and the rank order preference of *Inspired* relative to other familiar smoking cessation aids.

**Methods:**

Twenty-eight treatment-seeking smokers participated in this study. Participants attended a single one-hour session in which they received an overview of the goals of the *Inspired* mobile game, practiced submitting breath carbon monoxide (CO) samples, and played representative levels of the game. Participants were then told that they could play an extra level, or they could stop, complete an outcome survey, receive payment, and be dismissed. A sign-up sheet requesting personal contact information was available for those who wished to be notified when the full version of *Inspired* becomes available.

**Results:**

Using binary criteria for endorsement, participants indicated that, assuming it was currently available and fully developed, they would be more likely to use *Inspired* than: any other smoking cessation aid (21/28, 75%), the nicotine patch (23/28, 82%), a drug designed to reduce smoking cravings (23/28, 82%), or a program involving attendance in training sessions or support group meetings (27/28, 96%). In the questionnaire, participants indicated that both the *Inspired* program (26/28, 93%) and the *Inspired* game would be “Fun” (28/28, 100%), and 71% (20/28) reported that the program would help them personally quit smoking. Fifty-eight percent of participants (15/26) chose to continue playing the game rather than immediately collecting payment for participation and leaving. Eighty-two percent of participants (23/28) signed up to be notified when the full version of *Inspired* becomes available.

**Conclusions:**

This was the first study to evaluate a game-based contingency management app that uses game-based virtual goods as rewards for smoking abstinence. The outcomes suggest that the completed app has potential to be an effective smoking cessation aid that would be widely adopted by smokers wishing to quit.

## Introduction

Worldwide, cigarette-smoking–related death and illness are leading public health concerns, resulting in an estimated seven million deaths each year [1]. There are almost one billion smokers worldwide [1]. Thus, smoking cessation is a critical, worldwide public health concern [2].

Many efficacious smoking cessation interventions have been developed, yet ample room remains for improvement. Each year, one-third to one-half of all smokers attempt to quit at least once [3], but the annual incidence of successful quitting is less than 5% [4]. Media campaigns and cessation programs can increase quit attempts and successful cessation rates, respectively, by a margin of nearly 50% [3,5]. Smoking cessation rates that are better than those obtained with a placebo are achieved with: physician advice; counseling by health professionals; a variety of cognitive-behavioral, social-influence, and motivation-enhancement cessation programs; and drug treatments, including nicotine replacement therapies (gum, patch, spray, lozenge, and inhaler), selected antidepressant therapies (eg, bupropion), and nicotinic receptor agonist therapy (varenicline) [5,6]. Nevertheless, nearly 80% of smokers who attempt to quit do so without the assistance of any of these approaches [7]. Half or more consider counseling and cessation programs ineffective, and over a third consider pharmacotherapy ineffective [8]. Most young smokers report they would never use any of these methods, other than the nicotine patch (which only 50% would use) [9]. Thus, three major weaknesses of current approaches to smoking cessation are: underutilization, lack of appeal to smokers who wish to quit, and in general, modest efficacy in supporting smoking cessation. Clearly, more appealing and more efficacious smoking cessation interventions are needed.

Contingency management (CM) is one of the most efficacious aids for initiating smoking abstinence [10-12]. Contingency management for smoking cessation consists of delivering rewards (typically financial) contingent on objective evidence of smoking abstinence (eg, low levels of the combustion product carbon monoxide (CO) in the exhaled breath). Unfortunately, the effectiveness of CM on a population level has been limited by several constraints leading to low adoption rates and shorter than optimal treatment durations. These factors include the cost of providing the cash or cash-equivalent rewards [13-16], the distances that must be traveled, and the time required for participation in supervised monitoring procedures at a clinic [13,15]. Monetary rewards for smoking cessation can range from $100-$500 per person for approximately two 12-week interventions [11,17,18]. The cost of these payments limits the feasibility of widespread CM adoption. Furthermore, the ongoing nature of these costs limits the acceptability of longer-term treatment or booster sessions that could otherwise extend program effects (by reducing relapse).

CM procedures require biochemical verification of abstinence because participants are much more likely to falsify self-reports when rewards are delivered contingent on abstinence [19]. Carbon monoxide (CO) is one method to biochemically verify abstinence; however, the half-life of CO is short (approximately 3-6 hours), requiring at least twice-daily check-ins to verify abstinence [20]. To address this barrier, Dallery and colleagues developed an efficacious CM intervention that is delivered over the internet [11,17,21,22], in which participants are provided with a breath CO monitor, and remotely record and submit video clips of themselves providing their breath CO samples. More recently, *mobile* CM for smoking cessation (where participants use the camera on their smartphone to record and submit the video clips) has been shown to be feasible, acceptable, and efficacious in supporting smoking abstinence [23-25]. However, even these internet and mobile CM interventions rely on monetary incentives to support abstinence; thus, cost remains a barrier to widespread dissemination.

To directly address the remaining barriers for widely disseminating CM (ie, cost and sustainability), we proposed to develop a mobile game–based CM intervention for smoking cessation. As with existing mobile CM for smoking cessation, participants would be provided with a breath CO monitor and required to remotely record and submit video clips of themselves providing their breath CO samples twice daily. In our proposed mobile app, the *monetary rewards* typically used to incentivize bio-verified abstinence will be replaced with in‑game virtual-good rewards that can immediately be used to help players meet game objectives. Virtual goods can be provided by software at essentially no cost, yet they can have significant economic and monetary value (as evidenced by the multi-billion-dollar market for game-based virtual items) [26,27]. This suggests that rewards in the form of in-game content may readily substitute for monetary rewards in a CM procedure, drastically reducing cost while maintaining efficacy. The game will have the benefit of maximizing reward potency by minimizing delays to the receipt of rewards for abstinence once they are earned, as participants can immediately “consume” the rewards in the game. The proposed game design operationally encourages social support for smoking abstinence by imposing group contingencies [28], such as assembling players into teams and providing a reward that is only obtainable if all, or a majority of members reach a specified smoking cessation milestone (eg, no smoking for 24 hours). Operationally this design leverages self-interest (in obtaining access to the team reward) to incentivize social support (through interteam messaging) for others’ smoking cessation. The app will also enable standard, nonincentivized social support (eg, “click here to send congratulations to player C for <meeting a cessation milestone>”).

We previously published the results of an online survey of smokers to assess the social validity of a mobile game–based CM intervention for smoking cessation which uses virtual goods, instead of money, as rewards [29]. From a sample of 235 smokers recruited through Craigslist (ranging in age from 18-64 years), 75% reported playing video games. Among most smokers, 78% reported playing social games (ie, played casual games online). This rate is slightly higher than the population at large. Approximately 73% of all smokers and 70% of all video game players reported that contingent access to virtual rewards in the place of money would motivate smokers to abstain. Additionally, 75% of those surveyed would recommend or use a treatment such as this if they knew someone who wanted to quit, or if they were trying to quit themselves [29].

With the support of these promising outcomes, we developed and evaluated a prototype of *Inspired* (working title), a mobile game–based CM intervention to promote smoking cessation. The goal of the current project is to assess the feasibility and promise of *Inspired* by having treatment-seeking smokers play several levels of a prototype of the game and then ask them for qualitative feedback through a survey. We also indirectly observed feasibility and promise by recording users’ decisions to play an optional, extra level of the prototype.

## Methods

### Participants

Participants were recruited online through Craigslist, a free classified advertisement service, and Facebook, a social networking website. One hundred and eight individuals responded to our advertisements, reporting that they were smokers and indicating when they would be available to come in for a one-hour prototype testing session. All participants met the prescreening qualification criteria; therefore, no one who expressed an interest was excluded for any reason other than failure to respond to emails (N=28; see Table 1 for participant characteristics).

The advertisement specified that Rowan University researchers were seeking cigarette smokers to test a prototype of a game to help people quit smoking, and that they would be compensated $40 for their participation. Participants were eligible if they reported smoking cigarettes, expressed a desire to quit smoking, and were available during the testing session times. All study procedures were approved by the Rowan University Institutional Review Board. All participants provided informed consent before beginning the session.

### Materials and Procedure

#### Prototype/Demo

Because this was a prospective assessment of a planned intervention, a complete product was not yet available for evaluation in this study. Instead, led by one of the project investigators, subjects participated in a guided “walk-thru” of the proposed intervention in which they had hands-on experience utilizing the key components of the intervention that had been developed to date (“demo”), and were presented with mock-ups of planned features and when and where they would otherwise appear in the normal sequence of events in a fully developed version of the product.

Specifically, participants experienced recording and submitting breath CO readings, using a piCO+ breath carbon monoxide monitor (Bedfont, United Kingdom). In addition to submitting an initial breath CO sample, participants were also asked to imagine scenarios in which they had passed or failed various breath tests for a period and were told what rewards they would or would not have received given each scenario.

**Table 1:** Participant demographics (N=28).

Variable		Value
**Gender, n (%)**		
	Female	10 (36)
**Race, n (%)**		
	White	10 (36)
	Black	11 (39)
	Asian	1 (4)
	Unknown	6 (21)
**Ethnicity, n (%)**		
	Hispanic	5 (18)
	Not Hispanic	19 (68)
	Unknown	4 (14)
**Cigarettes per day, n (%)**		
	10 or less	16 (57)
	11-20	10 (36)
	21-30	2 (7)
**Fagerström Test for Nicotine Dependence, mean (SD)**		3.39 (2.6)

**Figure 1:**
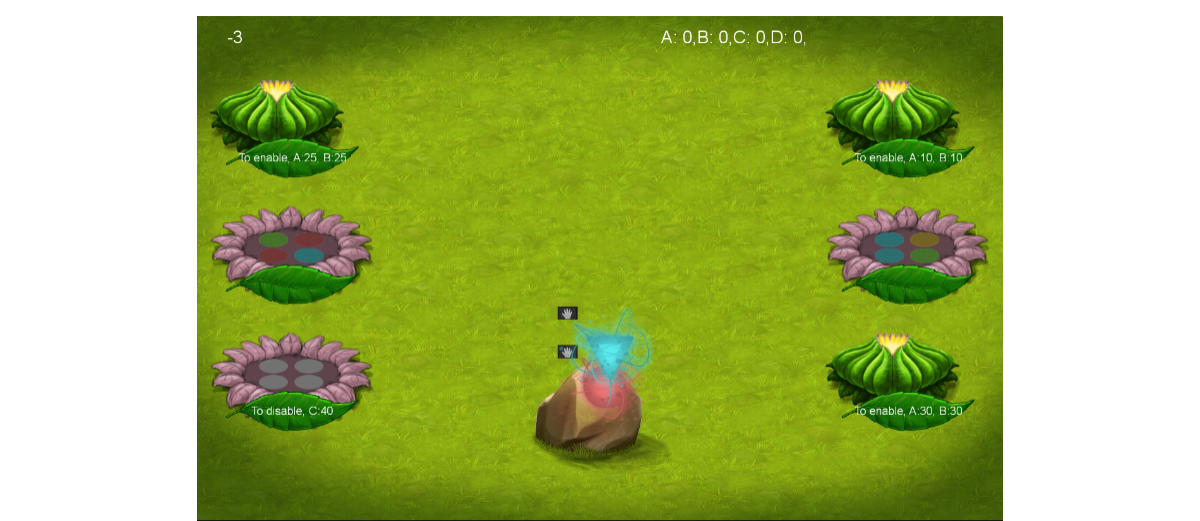
Screenshot of core game activity.

**Figure 2:**
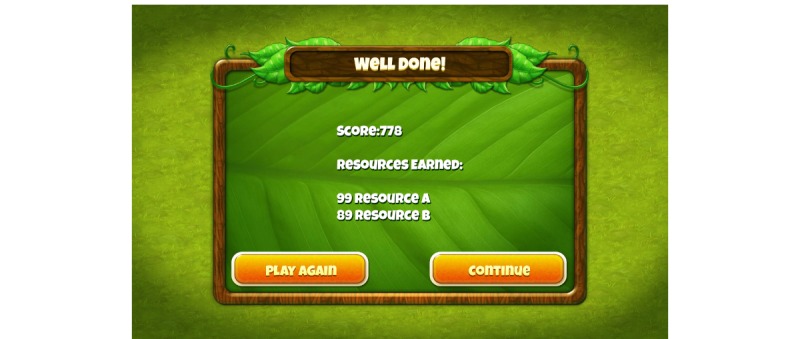
Screenshot of virtual rewards.

The demo version of the game that participants played illustrated the “core game experience.” The abstract objective of the game was “growth.” The core game experience was set in a lush vegetative environment, and the activity involved holding the tablet with both hands in landscape view and swiping different colored pollen-gems from a rotating queue center-screen into specific locations on lotus flowers to the left or right of the screen (see Figure 1). The challenge in this activity was to match color patterns under the pressure of time where the lotus flowers would eventually expire. The colored pollen-gems would also only remain in the queue for a limited time before being replaced. Better performance led to more lotus flowers being available within a level. At the end of the level, participants could see a hypothetical number of resources earned for completing that level (see Figure 2), which were awarded for completing sets, according to the difficulty of making the set (the least to most points were awarded as follows: no pattern < all same color < 4/4 matched the color template on the lotus flower). The levels became increasingly difficult because of the speed of the falling pollen and the difficulty with making four out of four matching sets. Each level was designed for casual gameplay, lasting approximately five minutes.

**Figure 3:**
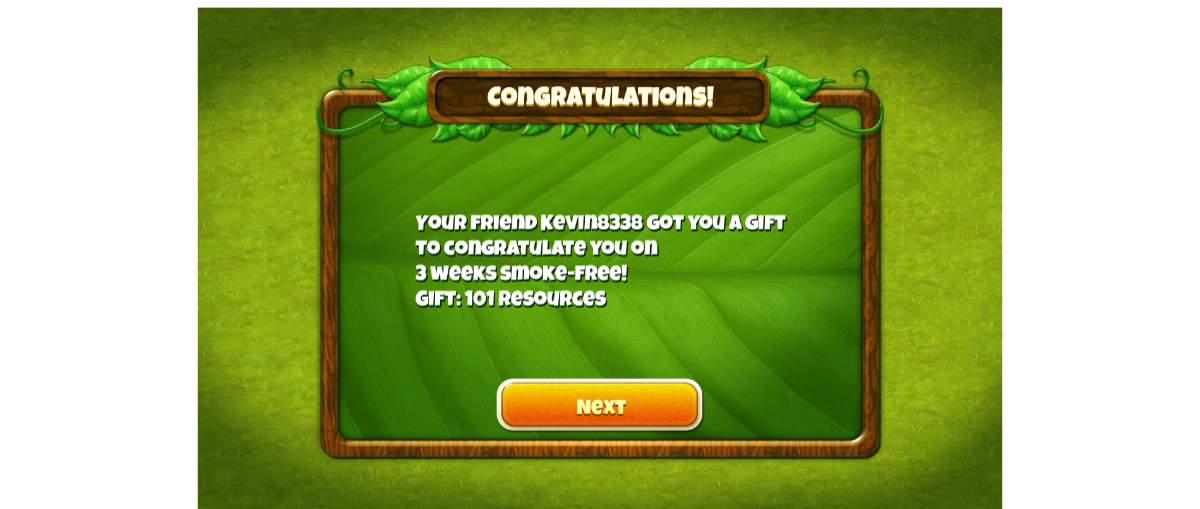
Screenshot of receiving a social reward.

**Figure 4:**
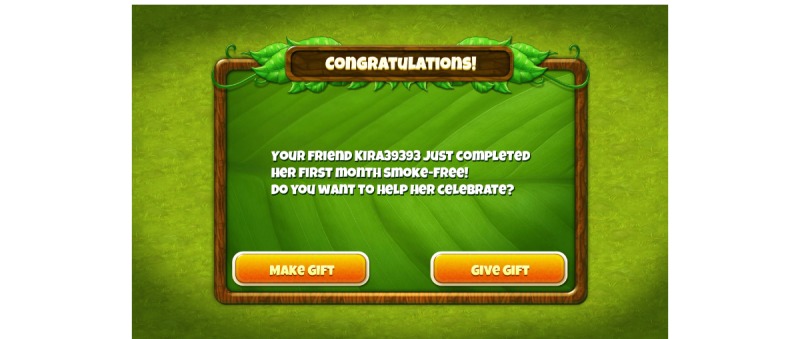
Screenshot showing how to give a social reward.

**Figure 5:**
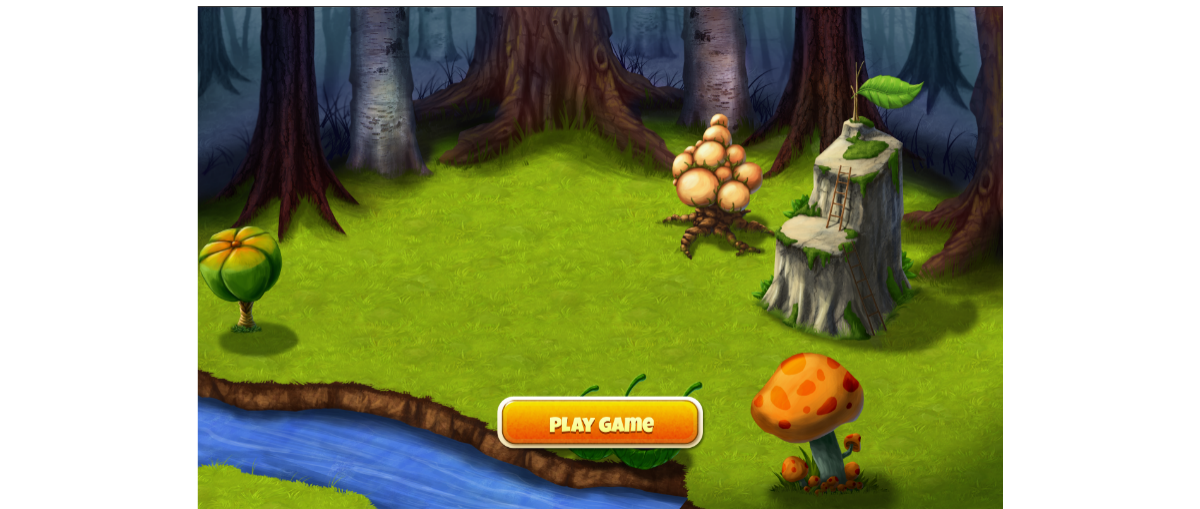
Screenshot of home screen early in game.

**Figure 6:**
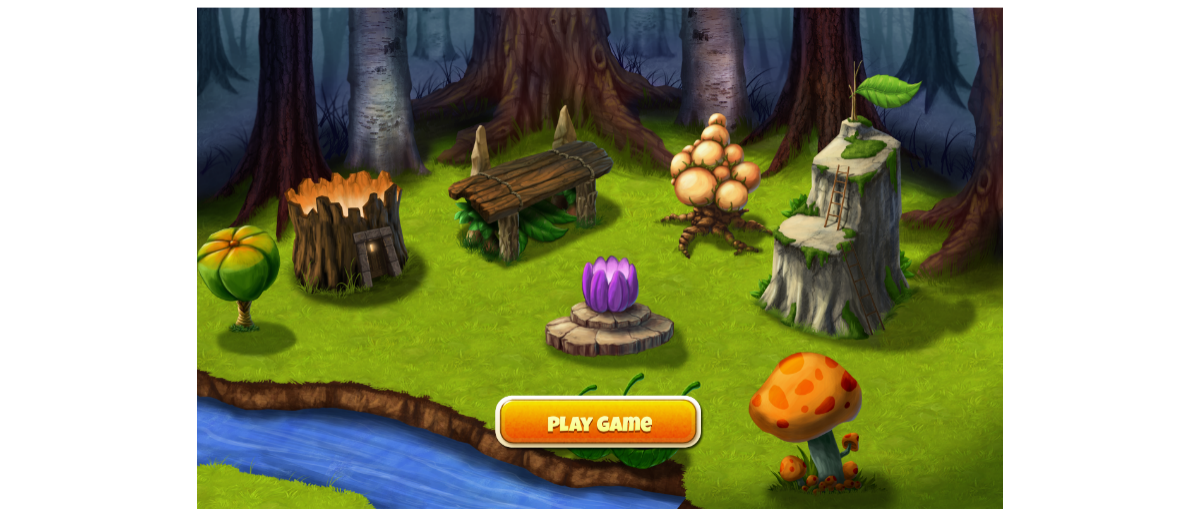
Screenshot of home screen later in game.

Features that were mocked-up or only verbally described included: push-notifications on the platform device (eg, “It’s time to take a breath test”); player-to-player messaging; gifting and social support for smoking cessation (players could “send” gifts on the gift screen, but receipt of the gifts by another player was not implemented, Figures 3 and 4); group contingencies; specific rewards for not-smoking (eg, a side-cache of various pollen-gems that persisted until used, or resources to build structures); and a requirement to submit a breath sample (pass or fail) to unlock access to the next game level. Players were also shown that there would be growth of structures displayed on the home page as they progressed through the game over time (in step with ever increasing abstinence through the course of the intervention; Figure 5 versus Figure 6), but they were instructed that in the full version of the game the structures would produce different virtual resources that the player could then utilize in core game play, to advance their progress in the game, to make and exchange gifts with other players, and more.

#### Study Participation Sessions

Seven groups (n=3-7 participants per group) of treatment-seeking smokers participated in the prototype evaluation sessions. Each group participated in one session, lasting approximately one hour. Sessions began with the consent process. Participants were then told how smoking releases carbon monoxide (CO) into the lungs as a by-product of combustion, and how our intervention could reliably detect if they had been smoking by measuring CO in the exhaled breath. A brief presentation described how the app would be designed to integrate breath monitoring with gameplay and in-game rewards to function as a smoking cessation aid. Each participant was loaned an Android tablet (Nexus 7 2013; Google, Asus) and a piCO+ CO meter (Bedfont; Kent, United Kingdom) and were asked to submit a video sample of their CO using the camera on the tablet. The video samples showed them exhaling into the CO meter and showing their CO value to the camera. All prototype game evaluation sessions were video recorded using a Samsung HMX-F90 camcorder.

One of the authors and game developers (NF) provided an overview of the game objectives to players, after which they were asked to begin playing the game on the Android tablet. Participants could ask questions about the game at any time. When subjects completed all the levels scheduled for use in the demonstration, they were told that there was one extra level that they had the option to play. They could either end the game at that point and finish the last few steps of the study (ie, complete an exit survey and receive payment), or they could stay a few minutes longer and play one extra level before completing the final steps. It was made clear that the choice was entirely theirs and that there would be no penalty for skipping the extra level.

At the end of the prototype evaluation session, participants completed a brief prototype evaluation survey, which consisted of four parts. The first part asked participants to classify how well they agreed with each item using a 100-mm visual analog scale (VAS; anchors, 0 mm= “Definitely Not,” 50 mm=“Maybe,” and 100 mm=“Yes, Absolutely”). The second part consisted of multiple-choice and free-response questions (eg, how much they would be willing to pay to use the program, what did they like most and least about the game, and more). The third part of the survey consisted of multiple-choice demographic questions to collect information about race, ethnicity, and gender. The fourth and final part of the survey was comprised of the six-item Fagerström Test for Nicotine Dependence [30].

Before leaving, participants were told that a sign-up sheet was available on which they could provide their contact information if they were interested in being contacted when the full version of the game was released.

### Data Analysis

The median and interquartile range (0-100 mm) for each item on the outcome evaluation were calculated. Additionally, the percentage of participants who “endorsed” each statement was defined in the following two ways: (1) ratings of 51 or higher on the VAS translate as an endorsement on a binary scale (eg, anchors “Disagree, Agree”), whereas (2) ratings of 67 or higher translate as an endorsement on a three-choice scale (eg, using the anchors “Definitely Not,” “Maybe,” and “Yes, Absolutely”).

## Results

Twenty-eight individuals participated in the prototype evaluation study (see Table 1 for demographic information). To analyze the VAS scores, prototype evaluations and their median (interquartile range, IQR) scores are presented in Table 2.

When an endorsement was defined by a ranking of 51 or higher (binary), at least 71% of participants endorsed statements that they would use *Inspired* (Q1)*,* recommend *Inspired* to a friend (Q2)*,* expect *Inspired* would help themselves (Q3) and others (Q4) quit smoking*,* consider both the game (Q6) and the intervention as a whole (Q5) to be “fun,” and that they would be more likely to use *Inspired* than any other smoking cessation aid they were familiar with (Q10a-e), including the nicotine patch (Q10b_,_ range 71%-100% , n=20-28, depending on the item; see Table 2).

**Table 2:** Prototype evaluation median (interquartile range, IQR) of visual analog scale (VAS) ratings and percent of endorsements at rankings of ≥51 (binary) and ≥67 (trinary; all % out of 28 total participants).

Item #		Median (IQR) VAS	Endorsed, % (≥51)	Endorsed, % (≥67)	Item
Q1		84 (67-98)	86	75	Would you use the proposed full version of *Inspired* to help you quit smoking and stay smoke-free?
Q2		95 (79-99)	93	89	Would you recommend *Inspired* to a friend that wants to quit smoking?
Q3		63 (50-94)	71	46	Do you think using the *Inspired* program would help you to quit smoking?
Q4		93 (69-98)	93	79	Do you think the *Inspired* program could help some smokers quit smoking?
Q5		86 (65-98)	93	71	Do you think using the *Inspired* program as a whole (including breath monitoring, playing the game, and giving and receiving rewards for not smoking) will be FUN?
Q6		86 (72-98)	100	79	Do you think the *Inspired* game will be FUN to play?
Q7		62 (51-68)	79	29	In terms of FUN, where do you think you would rank the *Inspired* game compared to all other games you have ever played on a smartphone (including games you played only once)?
Q8		70 (51-98)	75	50	Do you think incorporating information about the health benefits of not smoking directly into the game would make the *Inspired* program more effective in helping people quit smoking?
Q9		76 (69-99)	100	79	Do you think incorporating tips about how to quit smoking such as ways to deal with cravings directly into the game would make the *Inspired* program more effective in helping people quit smoking?
**Q10**					If the full version of *Inspired* were currently available, and you were selecting a smoking cessation aid to use in your next attempt to quit smoking, do you think you would be more likely to use *Inspired* than...
	Q10a	72 (51-97)	75	54	…any other smoking aid?
	Q10b	92 (59-98)	82	71	…the nicotine patch?
	Q10c	98 (59-100)	82	68	…a drug designed to help reduce your cravings?
	Q10d	92 (74-99)	96	86	…a program that involves you attending multiple training sessions or support group meetings?
	Q10e	87 (59-100)	93	68	…hypnosis?

The items with the lowest percentages of endorsement were Q3 (would help me), Q8 (add health benefit tips), and 10a (1st choice, 21/28, at least 75%), and the highest percentages of endorsement were Q6 (fun), and Q9 (add tips about cravings: 28/28, 100%). Alternatively, if endorsement was defined as a ranking of 67 or higher (trinary), at least 29% of participants (8/ 28) endorsed every item, at least 50% (14/28) endorsed all but two items (Q3 and Q7), and at least 75% (21/28) endorsed all but six items (Q3, Q7, Q8, Q10a, Q10c, and Q10e), with a range of at least 29%-89% (n=8-25) across all the items. The item that received the lowest percentage of endorsements was Q7 (fun relative to other games: 8/28, 29%), whereas the item that received the highest percentage of endorsements was Q2 (would recommend to a friend: 25/28, 89%). Additionally, when asked a multiple-choice question whether the game was fun, 63% (17/27) said “Yes,” 22% (6/27) said “Maybe, it has the potential to be fun,” and 15% (4/27) said, “No.” When asked the question, “If the *Inspired* program had been demonstrated to be just as effective as other smoking cessation aids (such as the nicotine patch), and included ongoing access to the game, the monitoring program, and a CO monitor that was yours to keep, how much would you be willing to pay for the program?”, the mean (SD) responses were $14.40 per month (SD $16.20) or $133 for a one-time purchase (SD $186).

In the free-response portion of the survey, when participants were asked what they liked best about the *Inspired* game, the most frequent response was that they liked the game itself, either because it was fun, creative, challenging, or because they liked the puzzle style of the game (12/28, 43%). Participants also reported that they liked the rewards delivered for abstinence (8/28, 29%), the community and social support aspects of the game (7/28, 24%), the simple instructions (5/28, 19%), the graphics (4/28, 14%), and the ability of the game to serve as a distraction from smoking (4/28, 14%). A couple of participants also mentioned that they liked the CO monitor (3/28, 10%). When asked what they liked least, the most frequent response was that they thought the game lacked variety (8/28, 29%). Participants also noted that glitches with the game needed to be resolved (eg, swiping gems to the correct location, screen loading, and more; 4/28, 14%), the graphics could be improved (4/28, 14%), and that the game was too easy (3/28, 10%). Other comments included: the CO meter was too bulky, there were too many screens, the game was too challenging, they did not like the idea of social support, and that the game felt disconnected from the rewards. Two participants did not indicate any weaknesses with the proposed intervention (2/28, 7%). Participants were also asked to give suggestions about how to move forward with the game, and the only response that appeared more than once was to improve variety in the game (9/28, 33%). Other suggestions were to provide real rewards for abstinence, resolve glitches, improve graphics, and to explore having insurance companies cover the cost of the game.

Fifty-eight percent of participants (15/26) given the option to play an extra level of the game chose to do so, and 86% (23/28) signed up to be notified when the full version of the game was released.

## Discussion

This was the first evaluation of a mobile game–based CM intervention for smoking cessation. The prototype of *Inspired* was endorsed on multiple dimensions by a group of treatment-seeking smokers. Most participants reported that they felt the game would help them, or a friend, quit smoking. For *Inspired* to be effective at motivating smokers to quit using game-based rewards as incentives for abstinence, it is critically important that the game be fun. If the game is not fun, the virtual rewards will not be effective at reinforcing abstinence. In the current study, the extent to which the prototype of *Inspired* was fun was evaluated in multiple ways. To begin, VAS responses in the prototype evaluation survey (see Table 2), addressed whether participants thought the *Inspired* intervention program was fun (Q5), and 71%-93% (n=20-26) of participants agreed, depending on how an “endorsement” was defined. Additionally, 82% (23/28) of participants reported that the game was either already fun or had the potential to become fun with further development. Finally, probably one of the strongest indicators that the game has potential to be both fun and effective at supporting smoking cessation was the behavior of participants when they were given the option to play an extra level. More than half of participants (16/ 28, 58%) decided to play the extra, optional level, which meant they may have delayed smoking their next cigarette (following about one hour of abstinence; ie, the duration of the study), as well as getting paid, by at least five additional minutes. The goal of the game is to decrease smoking, and the fact that the prototype for *Inspired* may have been capable of displacing smoking for even a brief period is encouraging. Between 57%-76% of participants (n=16-21) said they were more likely to use *Inspired* than any other smoking cessation aid, including evidence-based pharmacological interventions such as varenicline and the nicotine patch, which are endorsed in the Clinical Practice Guidelines for smoking cessation [6]. This finding supports previous research suggesting that the use of pharmacological or other evidence-based interventions may be not be preferred among individuals attempting to quit [8,9]. It should also be noted that mobile game–based smoking cessation is not incompatible with these other interventions but could instead serve as a fun, alternative, and yet evidence-based complement to these existing interventions.

Item Q7 on the prototype evaluation survey was included to help inform the game design team of how they were doing, at this early stage of development. Participants were asked to rank the intervention game relative to every other game they had ever played on their smartphone. Because participants were asked to compare this prototype of the game, which was less than 10% developed at the time of testing, to existing and fully developed mobile games, a median VAS of 64 was a promising outcome. Furthermore, the question asking how much participants would be willing to pay for the intervention suggests that there may be commercial viability of the proposed game, with answers ranging from $1.99-$60 per month, or $9.99-$700 as a one-time fee. Finally, 85% (23/27) of study participants signed up to be notified when *Inspired* becomes available so they can use it if they have not yet successfully quit smoking by that time, further supporting the potential commercial viability of the game.

Participants provided useful feedback for moving forward with game development, probably the most consistent of which involved adding variation to the game to keep it interesting and engaging. Overall, feedback about the type of game, the game graphics, as well as the social elements and CO monitoring in the game, were viewed favorably. Smaller, portable versions of the CO monitor have come to market since this prototype evaluation was conducted, thereby addressing concerns about the meter being bulky (eg, CO by Bedfont).

It should be noted that *Inspired* was designed to address smoking cessation specifically; therefore, multiple game design decisions were made to address the unique needs of individuals trying to quit. First, the core game mechanic, which required players to hold the device with both hands in landscape view and swipe pollen-gems into locations on various lotus flowers (see Figure 1), was chosen to make it difficult to simultaneously smoke while playing the game. Second, each level of the game was designed for casual gameplay, lasting approximately five-minutes, to reflect how long it might normally take to smoke a cigarette [31]. Third, visual elements of the game motif were associated with wellbeing and growth. The design intentionally avoided anything that might serve as a cue for smoking (eg, smoke, certain words, and more). This was done to avoid having the game elicit cue-induced cravings and subsequent smoking, and to enhance the ability of the game to displace smoking. Although not asked to comment on this directly, participants were asked to indirectly address this decision with Q8 and Q9 on the survey (see Table 2), where they reported the mobile game would be stronger if it incorporated messages about the health benefits of smoking cessation and tips about avoiding cravings. High endorsements on these two items suggested that future iterations of the game should explore incorporating this information in to the game, but in a way that does not also elicit cravings or trigger smoking.

The current study has a few limitations worth noting. First, because of the small sample size it was not possible to determine whether there were differences in endorsements, or other measures, between high and low nicotine dependent participants. Some items seemed to suggest differences, but it was not possible to evaluate the potential differences using inferential statistics. Second, information about the participants' individual histories with playing games, particularly mobile games, was not collected. Anecdotally, it was made clear that there was a range of past experiences. However, because the game is being designed as a smoking cessation aid, a level of heterogeneity of past experiences with video games is expected among the target population of treatment-seeking smokers as well. A third limitation is the possibility that participants rated the game favorably to avoid offending the experimenters and game designers (ie, demand characteristics). To mitigate this concern, we made it clear to participants at the beginning of the group sessions that they were being asked to give an honest evaluation of a very early version of the game, and that their feedback could help shape the future development of the game. Although it is impossible to rule out potential bias, participants felt comfortable giving the game a low rank-order relative to other, commercially available games (Q7 received the lowest scores), as well as other available smoking cessation aids. In the free response section participants provided useful feedback for improving the program. Finally, the fact that over half of the participants voluntarily played an extra, unrequired level of the game alleviates some concerns about bias; however, it cannot be ruled out.

Although there are other digital games that have been evaluated for smoking cessation, none are based on the empirically supported procedures and theoretical foundations of contingency management and behavior analysis [32-34]. This study is the first to show that a mobile game–based CM intervention has potential to be both helpful and fun to smokers who wish to quit. The prototype evaluation suggests that the proposed game would not only reduce the cost of delivering CM for smoking cessation and enable extended program reach and duration, it might also be preferred over currently extant smoking cessation aids and interventions.

## Abbreviations

CM: contingency management
CO: carbon monoxide
IQR: interquartile range
VAS: visual analog scale
